# Unveiling final-year students and new graduates’ concerns, preparedness, and learning experiences during the pandemic in Qatar: A cross-sectional survey

**DOI:** 10.1016/j.heliyon.2023.e22337

**Published:** 2023-11-19

**Authors:** Yasin M. Yasin, Albara Alomari, Wilma ten Ham-Baloyi, Samaneh Alinejad Mofrad, Lorna J. Moxham, Elizabeth J. Halcomb, Ritin Fernandez

**Affiliations:** a University of Doha for Science and Technology, P. O. Box 24449, Doha, Qatar; b Nelson Mandela University, South Africa; c Mashhad University of Medical Sciences, Iran; d University of Wollongong, Australia; e University of Newcastle, Australia

**Keywords:** Pandemic-era, COVID-19, Educational experiences, Clinical preparedness, Worries, nursing students

## Abstract

**Background:**

The emergence of COVID-19 interrupted education worldwide and educational institutions were forced to switch to an online learning (E-learning) environment.

**Objectives:**

To explore the perceived educational experiences, worries, and preparedness to enter clinical practice among final-year nursing students and new graduate nurses after studying during the COVID-19 pandemic.

**Design:**

A cross-sectional survey design.

**Setting:**

and participants: An online survey of final-year undergraduate bachelor of nursing students at a single university in Qatar and new graduates across 14 health facilities in Qatar was undertaken between May 2022 and July 2022.

**Methods:**

Participant demographics, experiences of E-learning and perceptions of readiness for practice were collected using validated instruments and open-ended questions. Descriptive statistics and thematic exploration were used to analyze the data.

**Results:**

Fifty-nine final-year students and 23 new graduates with an average age of 23.95 years (SD = 3.89) responded to the survey. Face-to-face clinical placement learning was preferred by 50 % of students and 66.1 % of graduates. During COVID-19, the majority of the participants indicated that strategies like practice kits and online simulations were implemented. While 61 % of new graduates felt well-prepared in their nursing skills, and 87 % felt confident, less than half of the students still in their final year of education felt prepared or confident. Increased stress and a perceived impact on education quality were reported due to changes in learning modalities. Despite these challenges, 81.1 % of students and 95.7 % of graduates felt they had developed sufficient professional values, with most looking forward to becoming registered nurses. The qualitative themes that emerged related to 1) adjusting to online learning, 2) experiencing restrictions in clinical learning skills, 3) feeling less confident and 4) experiencing increased stress.

**Conclusions:**

Despite experiencing emotional and educational challenges, the participants in this study felt that their education prepared them for clinical practice. Innovative strategies and unique educational experiences used by educators enhanced participants' clinical skills and readiness for practice.

## 1 Introduction

The health crisis emanating from COVID-19 not only affected the front-line workforce and healthcare leaders but also all educational institutions and communities globally [1]. Educational institutions were forced to close to achieve mandatory social distancing [2]. In response to the pandemic, global nursing bodies responded swiftly and comprehensively to address the public's health needs, provide resources for nurses, and adapt educational methods to mitigate risk, all the while confronting financial, technological, academic, and emotional challenges [3]. Nursing schools faced the necessity to shift the delivery of nursing education to online methods to prepare the next generation of healthcare providers. Particularly, significant concerns regarding the safety of student nurses played a pivotal role in shaping this decision [4].

In response to the shift in education delivery, nursing schools employed innovative strategies to sustain clinical training. Simulations, virtual reality experiences, and telehealth scenarios emerged as prevalent strategies employed to emulate real-world clinical experiences [4]. In response to the pandemic, nursing education swiftly adapted by integrating online discussion platforms to support case studies and problem-solving activities, thereby ensuring continued peer interaction and collaborative learning [5]. Additionally, online simulations were adopted to address the limited availability of clinical practice opportunities [6].

Given the rapid changes, nursing students faced numerous concerns surrounding their education during the COVID-19 pandemic. These included anxiety over transitioning to online learning, uncertainty about meeting clinical objectives, and worries about acquiring necessary practical skills [7]. The fear of academic delays and the potential impact on future career prospects further added to their stress [1]. These concerns were heightened by the challenging nature of nursing, which traditionally requires substantial hands-on training in a clinical setting.

Nursing students, had to face the added challenges of meeting clinical objectives in an online learning environment, which was a unique experience for them [8]. Following their studies during the pandemic, the readiness of nursing students for clinical practice became a point of significant concern. While e-learning methods and alternative training strategies were implemented, the question remained whether these measures sufficiently prepared students for clinical practice [9]. The long-term effects of these educational adaptations on the preparedness and competency of nursing graduates entering the healthcare workforce will be a critical area of investigation in future studies.

In Qatar, similar to other countries, final-year nursing students and recent graduates encountered multiple distinctive challenges during the COVID-19 pandemic. One notable challenge was the abrupt surge in cases, which imposed a burden on the healthcare system [10]. The hospitals were compelled to allocate their personnel to the new COVID-19 facilities, leaving the new graduate students without the necessary space and mentors to train them in clinical placements [11]. This could present a significant challenge to their readiness and confidence in dealing with complex cases, as they were thrust into demanding situations. Additionally, Qatar's diverse patient population presents communication challenges. Patients hail from a variety of cultural backgrounds, making it crucial for students to swiftly adapt to culturally sensitive care practices. A certain difficulty that final-year students and recent graduates encountered during the COVID-19 pandemic in Qatar, unlike other regions, was the disruption of teaching and assessment. The occurrence of this disturbance caused the delay of the concluding assessments, potentially resulting in heightened levels of anxiety among the students [12]. A study of final-year students and new graduates in Qatar and other regions found that students in Qatar were more concerned about their academic future and their job prospects than students in other countries [13].

While numerous studies globally have begun to investigate the impact of COVID-19 on nursing education [4,7], a specific knowledge gap remains, particularly within the context of Qatar. The nursing profession, by nature, is hands-on, and it is pivotal to understand how the shift to e-learning affected students’ perceived readiness for actual clinical practice, confidence in practice, concerns, and strategies employed during the pandemic, as well as the effects of the swift shift to e-learning. Little is known regarding the effectiveness of these strategies online versus face-to-face or in-person learning on clinical learning during a pandemic, which warrants further exploration. The aim of this paper was therefore to explore final-year nursing students and new graduate nurses perceived educational experiences, worries, and preparedness to enter clinical practice when studying during the COVID-19 pandemic. The findings will not only provide invaluable insights into the unique experiences of nursing students in Qatar during this global health crisis but will also enrich our understanding of the wider implications of the swift adoption of various innovative teaching strategies in nursing education and will be instrumental in shaping future educational strategies. This, knowledge in turn will strengthen the resilience of the nursing education system, making it more robust against similar disruptions.

## 2 Methods

### 2.1 Study design

A descriptive exploratory cross-sectional survey.

### *2.2* Participants

Participants for the study were final-year undergraduate nursing students in the Bachelor Nursing Regular Track (BNRT) at a university in Qatar and newly graduated nurses who started their Transition to Practice Program in 2022. The latter group comprised nurses with less than a year of professional experience post-graduation. A convenience sampling approach was employed in conducting this online survey due to its practicality and the expedient manner in which it enabled data collection from a pool of respondents.

### 2.3 Instrumentation

The survey tool was designed by the research team and delivered online via Survey Monkey (new graduate) and Qualtrics XM (final year students). The survey was structured into distinct sections to address various facets of the nursing education experience. It began with questions about sociodemographic information, such as age and gender. This was followed by separate sections focusing on the nursing program's delivery method, learning approaches, and the support provided by the university to facilitate learning. Additionally, the survey included a section with five open-ended questions designed to capture qualitative insights into the impact of COVID-19 on various aspects of the learning experience. These qualitative questions were strategically placed to collect immediate perceptions after respondents had considered specific areas of their education. The survey also gathered data on participants' concerns about how altered study environments might affect their academic progress, readiness for clinical practice, and confidence in their skills. The Altered Student Study Environment Tool (ASSET) was used to assess worries related to the influence of changed study environments on academic progress [14]. This 11-item scale uses a five-point Likert scale, ranging from 1 (strongly disagree) to 5 (strongly agree). In the initial study, an exploratory factor analysis supported a three-factor structure, encompassing attending clinical placement (α = 0.92), completing clinical placement (α = 0.77), and grade attainment (α = 0.80). The ASSET tool was incorporated into the section of the survey focusing specifically on concerns related to the impact of changed study environments on academic outcomes.

The 20-item Casey-Fink Readiness for Practice Survey (CFRPS) was used to evaluate participants' readiness and confidence in performing essential nursing tasks on a four-point Likert scale ranging from 1 (strongly disagree) to 4 (strongly agree) [15]. The CFRPS comprises four factors all with moderate to high reliability: Clinical Problem Solving (α = 0.80), Learning Techniques (α = 0.50), Professional Identity (α = 0.65), and Trials and Tribulations (α = 0.63). An additional three items were adapted from Confidence and Interest in Critical Care [16] which sought to gauge confidence in nursing practice after graduating from the nursing program during the COVID-19 pandemic. Expert opinion was employed to confirm content validity, while exploratory and confirmatory factor analysis were used to ascertain construct validity [17]. The CFRPS was part of the section in the survey that aimed to evaluate participants’ confidence and readiness for clinical practice.

### 2.4 Data collection

The university's Student and Enrolment Services emailed the study details and survey links to the nursing undergraduates. A Quick Response (QR) code was strategically stationed around the university premises and served as reminders for students to fill out the survey. A QR code is a two-dimensional barcode that can be scanned using a smartphone camera. Once scanned, it led participants directly to the survey and the participant information sheet. Concurrently, the new graduate nurses undergoing their Transition to Practice Program across 14 health facilities in Qatar were also invited to participate in the study. For this demographic, a QR code leading to the survey and participant information sheet was positioned at the entrance of the auditorium where they held their first gathering. The QR code was also displayed on the main screen during breaks in orientation sessions. Data collection occurred between May 2022 and July 2022.

### 2.5 Ethical considerations

Three Institutional Review Boards granted Human Research Ethics Committee approval for the study, with the following approval numbers: MRC-01-22-125, REB22-0287, and PHCC/DCR/2022/04/014. Participants were informed that by completing the survey, they were implicitly providing consent. They were also assured that their participation was voluntary and would not adversely affect their academic pursuits or employment.

### 2.6 Data analysis

#### 2.6.1 Quantitative data

All data were exported from the survey management software into the Statistical Package for Social Sciences (SPSS) version 28 for analysis. Missing values were replaced using the Expectation-Maximization algorithm. Descriptive statistics including frequencies, means, and standard deviations were calculated. This is a descriptive study and inferential statistics were not used in data analysis.

#### 2.6.2 Qualitative data

Responses to qualitative questions were entered into an Excel spreadsheet and responses for each question were categorized using a thematic analysis approach. Multiple readings by authors on an individual basis facilitated the identification of patterns. This process created open codes that were then assembled and assigned an initial brief description. This procedure supported the identification of preliminary themes. The coded extracts were consistently socialized within the research team until a consensus was reached on the final themes and the most appropriate nomenclature to describe the findings was agreed.

The authors adhered to the four trustworthiness criteria outlined by Schwandt et al. [18], which include credibility, confirmability, dependability, and transferability. The enhancement of credibility and confirmability was achieved through discussions among the authors on the analysis and emerging themes [18]. Credibility and transparency were promoted by providing participants with pseudonyms and quotes (Schwandt et al., 2007). The authors ensured dependability by providing a detailed description of the analytical process.

## 3 Results

Of the 82 participants who responded, 59 (72 %) were final-year students and 23 (28 %) were new graduates. Most participants (84 %) were female with the average age of all participants being 23.95 years (SD = 3.89). More than 60 % of both students and new graduates reported that prior to the COVID-19 pandemic, the most frequent form of course delivery for the theoretical component was face-to-face and approximately 50 % preferred that method for learning theory. For clinical learning, the majority of students (66.1 %) and new graduates (91.3 %) preferred clinical placement in a healthcare environment. Less than a quarter (20.3 %) of students preferred face-to-face simulation (Table 1).

**Table 1:** Participant Demographics and preferred mode for Learning.

		Students (n = 59)	New graduates (n = 23)	All (n = 82)
		**Mean (SD)**	**Mean (SD)**	**Mean (SD)**
Age		23.78 (4.12)	24.39 (3.26)	23.95 (3.89)
		**n (%)**	**n (%)**	**n (%)**
Gender	Male	8 (*13.6*)	3 (*13*)	11 (*13.4*)
	Female	49 (*83.1*)	20 (*87*)	69 (*84.1*)
Mode of course delivery pre COVID-19	Fully face to face	37 (*62.7*)	14 (*60.9*)	51 (*62.2*)
	Fully Online	5 (*8.5*)	1 (*4.3*)	6 (*7.3*)
	Hybrid	17 (*28.8*)	8 (*34.8*)	25 (*30.5*)
Preferred delivery mode: theory courses	Fully Face-to-Face	30 (*50.8*)	13 (*56.5*)	43 (*52.4*)
	Fully Online	7 (*11.9*)	2 (*8.7*)	9 (*11*)
	Hybrid	22 (*37.3*)	8 (*34.8*)	30 (*36.6*)
Preferred delivery mode: clinical courses	Placement in a healthcare setting	39 (*66.1*)	21 (*91.3*)	60 (*73.2*)
	Face-to-Face Simulation	12 (*20.3*)	2 (*8.7*)	14 (*17.1*)
	Online and Face-to-Face	6 (*10.2*)	–	6 (*10.2*)
	Computerized Simulation	1 (*1.7*)	–	1 (*1.7*)
	Other	1 (*1.7*)	–	1 (*1.7*)

### 3.1 Strategies to assist with clinical learning during the Covid-19 pandemic

The majority of students (78 %) and more than half (56.5 %) of new graduates indicated that they were provided with practice kits to assist their clinical learning. Over three-quarters (78 %) of the participants reported they had access to simulation. Access to online live chats was reported by 27.1 % and 39.1 % of students and new graduates respectively. Sixty-one per cent of students and 87 % of new graduates reported having online demonstrations of clinical skills. The use of virtual reality to support clinical learning was reported by 30.5 % of students and 52.2 % of new graduates respectively. Less than a quarter of the students (20.3 %) and half the new graduates (47.8 %) reported having increased access to staff to assist clinical learning during the COVID-19 pandemic (Table 2).

**Table 2:** Strategies to assist with clinical learning during the COVID-19 pandemic.

**Strategy**	Students (n = 59)	New graduates (n = 23)	All (n = 82)
	n (%)	n (%)	n (%)
Providing practice kits (e.g., wound care/suturing) (Yes)	46 (78)	13 (56.5)	59 (72)
Simulation experiences (Yes)	46 (78)	18 (78.3)	64 (78)
Online live chats (Yes)	16 (27.1)	9 (39.1)	25 (30.5)
Online demonstration of skills (Yes)	36 (61)	20 (87)	56 (68.3)
Virtual reality (Yes)	18 (30.5)	12 (52.2)	30 (36.6)
Increased access to staff (Yes)	12 (20.3)	11 (47.8)	23 (28)

### 3.2 Preparation to work as a registered nurse

More than half of the students (56 %) felt their nursing education somewhat prepared them and 61 % of new graduates felt well-prepared to provide patient care. Over half of the students (56 %) felt that the COVID-19 pandemic had a high impact on their overall nursing education, however, only 21.7 % of the new graduates felt the same. The majority of the students (81.1 %) and the new graduates (95.7 %) indicated that they developed sufficient professional values to be competent Registered Nurses (Table 3).

**Table 3:** Preparation and Confidence to work as a Registered Nurse.

	Response	Students (n = 59)	New graduate (n = 23)	All (n = 82)
		n (%)	n	n
***Preparation to work as a Registered Nurse***				
Nursing education preparing/prepared you to provide patient care	Not at all prepared	1 (1.7)	–	1 (1.2)
	Somewhat prepared	33 (55.9)	2 (8.7)	35 (42.6)
	Well prepared	22 (37.3)	14 (60.9)	36 (43.9)
	Extremely well prepared	3 (5.1)	7 (30.4)	10 (12.2)
COVID-19 pandemic impact on your overall nursing education	No or minor impact	5 (8.5)	5 (21.7)	10 (12.2)
	Moderate impact	21 (35.6)	13 (56.5)	34 (41.5)
	High impact	33 (56)	5 (21.7)	38 (46.3)
Do you feel you are developing sufficient professional values to be a competent Registered Nurse?	Yes	48 (81.1)	22 (95.7)	70 (85.4)
	No	3 (5.1)	1 (4.3)	4 (4.9)
	Unsure	8 (13.6)	–	8 (9.8)
***Confidence to work as a Registered Nurse***				
Do you feel your undergraduate studies is preparing you to work as a Registered Nurse?	Yes	45 (76.3)	23 (100)	68 (82.9)
	No	4 (6.8)	–	4 (4.9)
	Unsure	10 (16.9)	–	10 (12.2)
Do you feel you have sufficient skills to be a competent Registered Nurse?	Yes	28 (47.5)	20 (87)	48 (58.5)
	No	9 (15.3)	0	9 (11)
	Unsure	22 (37.3)	3 (13)	25 (30.5)
Do you feel you have sufficient knowledge to be a competent Registered Nurse?	Yes	26 (44.1)	17 (73.9)	43 (52.4)
	No	14 (23.7)	0	14 (17.1)
	Unsure	19 (32.2)	6	25 (30.5)

### 3.3 Confidence to work as a registered nurse

More than three-quarters of the students (76.3 %) and all new graduates felt that their undergraduate studies prepared them to work as Registered Nurses. However, less than half of the students (47.5 %) and 87 % of new graduates felt that they had sufficient skills to be a competent Registered Nurse. Similarly, less than half of the students (44.1 %) and 73.9 % of new graduates felt that they had sufficient knowledge to be a competent Registered Nurse (Table 3).

### 3.4 Worries about the study environment

For students, the overall mean score for concerns during the COVID-19 pandemic was 3.8 (SD = 0.59). The mean scores for the subscales attending clinical placement, completion of clinical placement and grade attainment were 3.37 (SD = 1.03), 4.21 (SD = 0.66), and 3.71 (SD = 0.72) respectively.

For new graduates, the overall mean score for concerns as a student during the COVID-19 pandemic was 3.62 (SD 1.38). The mean scores for the subscales attending clinical placement, completion of clinical placement and grade attainment were 3.35 (SD = 1.77), 3.88 (SD = 1.72), and 3.59 (SD = 1.38) respectively (Table 4).

**Table 4:** Worries about the study environment.

	Students (n = 59)	New graduate (n = 23)	All (n = 82)
	Mean (SD)	Mean (SD)	Mean (SD)
**Worries about the study environment**			
Attending clinical placement	3.37 (1.03)	3.35 (1.77)	3.36 (1.27)
Completion of clinical placement	4.21 (0.66)	3.88 (1.72)	4.12 (1.06)
Grade attainment	3.71 (0.72)	3.59 (1.38)	3.68 (0.95)
Overall concerns	3.80 (0.59)	3.62 (1.38)	3.75 (0.87)
**Readiness for Practice**			
Clinical Problem Solving	2.86 (0.38)	3.18 (0.52)	2.95 (0.45)
Learning Techniques	2.81 (0.68)	3.02 (0.67)	2.87 (0.67)
Professional Identity	3.0 (0.46)	3.49 (0.50)	3.14 (0.51)
Trials and Tribulations	2.56 (0.37)	2.43 (0.43)	2.53 (0.39)
Overall readiness for practice	2.80 (0.3)	3.63 (1.36)	2.86 (0.35)

### 3.5 Readiness for practice

For students, the overall mean score for readiness to practice was 2.8 (SD 0.3). The mean scores for the subscales of clinical problem-solving, learning techniques, professional identity, and trials and tribulation were 2.86 (SD = 0.38), 2.81 (SD = 0.68), 3 (SD = 0.46) and 2.56 (SD = 0.37) respectively.

For new graduates, the overall mean score for readiness to practice was 3.63 (SD 1.36). The mean scores for the subscales of clinical problem-solving, learning techniques, professional identity, and trials and tribulation were 3.18 (SD = 0.52), 3.02 (SD = 0.67), 3.49 (SD = 0.50) and 2.43 (SD = 0.43) respectively (Table 4).

### 3.6 Qualitative results

The themes for both groups are remarkably similar. Data analysis revealed the impact that the COVID-19 pandemic had on participants’ overall nursing education related to 1) adjusting to on-line learning, 2) experiencing restrictions in clinical learning skills, 3) feeling less confident and 4) experiencing increased stress. Participants described how adjusting to on-line learning was very challenging. Long hours of virtual learning were considered taxing and adjusting to a different learning style stressful. One participant said, *“Moving from in-person classes to online classes increased my stress and anxiety level because I was easily distracted, less focused and less engaged during online classes”.* Participants were aware of their learning styles and lamented that their learning needs were not being met. This is illustrated by a participant saying,

“*I am a tactile-kinesthetic learner and I usually need the interaction of the physical environment and the classroom setting to facilitate my learning hence COVID-19 pandemic and being stuck at home-learning hindered my learning leading me to have decreased my focus and motivation”.*

In addition to changes in theoretical instruction, participants were unable to attend clinical placements to engage in clinical nursing skills or when they were on placement – patient interaction was restricted, and they were fearful. One participant stated “*more restrictions in clinical setting, especially with respiratory procedures. I was feeling scared to come in contact with patients”.* Another said*, “the pandemic has surely affected my overall experience since nursing is a hands-on profession, the lack of clinical placements at the time had a negative effect on clinical skills and clinical judgement”.* A participant said that they “*missed lab skills and clinical hours*” which led to them feeling “*less confidence, more anxiety, loneliness, and feeling overwhelmed*”.

Participants were invited to identify strategies they used to manage the impact of the COVID-19 pandemic on their overall nursing education. Three strategies emerged as those most often used. These were 1) being more conscious of time management, 2) changed learning patterns and 3) investing in their own mental and physical health. Managing time whilst being at home with the many distractions that can bring led students to say that they “*always make extra time to study, do my assignments earlier, meet the instructor more often through zoom meetings to ask questions”.* Participants said they changed their learning patterns which included having to “*study more by reading the books*” and engaging in “*self-studying, video calling with friends to have study sessions, and practicing my skills by roleplaying”.* The COVID-19 pandemic raised awareness of personal physical and mental health needs. Participants spoke about this in their responses saying that they made sure they developed “*a strong* support *system”,* that they *“take breaks in between whenever I am tired” and how they ensured that they had “good sleep, good diet”* and “*be optimistic*”.

Participants were asked to identify the top three strategies that they considered most helpful for their preparation to enter practice as a registered nurse. Analysis identified three themes 1) simulations, 2) practice kits and 3) increased access to staff. They spoke about how engaging in simulations through “*on-line demonstration of skills*” and experiencing “*virtual reality*” helped with learning. Education institutions pivoted to deliver learning to students and practice kits were sent to students as well as increasing access to instructors/staff which participants appreciated.

Final year participants reflected on what they felt would be the most challenging in terms of starting their career as a registered nurse. Overwhelmingly, responses indicated that they were most concerned about the transition, and the fact that they will be working more independently which meant more responsibility. The transition from student to RN was “*scary*. Thinking about having to “*Manage work-load and shifts*” be “*resilient*”, be able to be “*quick and have adaptive working habits*”, “*deal with ethical decision-making*”, and practice “*stress management*” were all considerations raised by the students. The move from student to RN was summed up with a student saying that it ultimately meant *“not being able to hide under the protection of ‘student’ designation*”.

In response to questions about support most participants indicated that family and friends were their main means of support. As one student stated, “*friends and family - having a strong support system really helped in coping in difficult situations*”. Some indicated that being engaged with the university was also a means of support. “*Instructors are always willing to support us”,* they are *“just an email away”, “their feedback and the materials provided for learning*”, were all considered supportive.

When asked about their opinion of becoming a registered nurse, final year nursing student said that *“I am looking forward to working as a registered nurse*”. However, few were “*skeptical about being a registered nurse*” and yet others said they “*did not know*” how they now felt about being a registered nurse.

## 4 Discussion

This study aimed to examine the perceptions of final year nursing students and new graduate nurses regarding their educational experiences, worries, and readiness to embark on clinical practice following their studies during the COVID-19 pandemic. Data were collected using an online survey over a two month period during the third wave of COVID-19 pandemic.

The main strategies that were reported by study participants to assist with clinical learning during the COVID-19 pandemic included online demonstrations of skills (to a lesser extent with new graduates), simulation experiences, and practice kits (to a lesser extent with new graduates). In our study, traditional face-to-face clinical learning strategies were replaced with alternative methods, which have been used globally during the COVID-19 pandemic. These new strategies offer benefits such as improved understanding of the subject matter and enhanced confidence gained through discussions and role-playing. However, they also come with drawbacks, including decreased concentration and challenges in maintaining academic integrity [19].

Both students and new graduates in our study indicated similar worries about the learning environment, especially related to attending clinical placement, completion of clinical placement and grade attainment. Similar results were found in other studies [20,21]. Managing these worries requires support through a structured learning environment, timely communication about changes or updates, tailored assessments according to the learning environment and extended grace. Additionally, providing coping mechanisms and interventions to assist students to continue their studies [20], especially as the COVID-19 pandemic was reported by both groups of study participants to have influenced the education experiences and may have limited their exposure to clinical practice [22].

The quantitative findings showed mixed educational experiences with students generally reporting more negative experiences compared to new graduates. Nursing and graduate education experiences during COVID-19 have been explored in other studies, with similar findings [22]. The study participants indicated various positive experiences related to equipping them to deliver patient care, being able to qualify to work as a registered nurse and possessing adequate professional values to be a competent nurse. These findings are similar to other studies which found that despite negative educational experiences during the pandemic, resilience strengthened a desire to become a nurse and provided unique opportunities of learning [23]. Although not measured in this study, resilience could have influenced these positive experiences, but requires further exploration.

The qualitative findings among nursing students revealed that overall, their education experiences during COVID-19 were mixed providing extra support for the quantitative findings. Participants expressed negative experiences such as challenges adjusting to online learning, restrictions in clinical learning skills, feeling less confident, experiencing increased stress, and concerns regarding transitions, as found elsewhere. However, they also identified strategies that enhanced their learning experiences related to time management, learning and mental and physical health.

New graduates compared to final year students generally reported higher levels of readiness, being better prepared to provide patient care as well as having sufficient skills and knowledge to be more confident to work as a registered nurse. Similar findings have been reported elsewhere [24,25]. These results could be due to graduate nurses receiving extensive support from the university to prepare them for practice as their final year prior to graduation was during the peak of the pandemic. Additionally, final year nurses compared to graduate nurses have less acquired work experience which has been found to be a significant predictor of readiness for practice, feeling prepared and having confidence in working as a qualified nurse [24]. To enhance nursing students' readiness for practice, preparedness and confidence to work as a registered nurse, high fidelity simulations, self-directed learning laboratory and reflective learning practices as well as guidance, support and instant feedback in the clinical setting have been recommended [26]. Furthermore, the qualitative results underscored the significance of simulations and practice kits in enhancing students’ readiness. Increased access to faculty was also highlighted as beneficial. Students generally expressed the importance of support throughout their journey, and the majority were eagerly anticipating their transition into registered nursing roles. These experiences were found elsewhere [8,25], and confirm most of the quantitative findings, particularly related to reported strategies to assist with clinical learning, worries about the study environment, positive experiences of being able to qualify to work as a registered nurse, as well as feeling less confident.

Further, the qualitative findings provide a deeper understanding regarding educational experiences during COVID-19 and can guide educators and preceptors to enhance these experiences. For example, to enhance educational experiences among nursing students, their preferences, needs, effective strategies as well as support when offering course delivery for both the theoretical and clinical components, should be considered, as recommended elsewhere [27]. Additionally, limitations in terms of lack of human and emotional interactions when using online or e-strategies for learning [28], should be considered as most study participants preferred face-to-face instruction for theory courses and favored clinical placement in a health care environment for clinical courses. Tailoring teaching strategies according to student needs, determining the educator and clinical preceptor's level of competency to provide these strategies in an efficient manner, and determining whether the clinical setting where students are placed can accommodate the implementation of these strategies, is therefore crucial [29].

Lastly, to improve educational experiences, during (future) pandemics, emergency education preparedness plans are required that promote effective teaching strategies tailored to the students’ needs, addressing the crucial role of preceptors and educators, while considering student mental health and wellbeing through collaborative partnerships between educational institutions and clinical partners.

### 4.1 Strengths and limitations

One of the key strengths of this study is its timely focus on an under-researched area, especially within the context of Qatar. The study's use of both qualitative and quantitative methods provides a well-rounded, comprehensive view of the subject matter. In addition, the use of validated instruments to collect data adds to the robustness of the study. Although this study has provided valuable information into final year nursing student and new graduate nurses' perceptions about their education experiences during the COVID-19 pandemic and preparedness for practice, some limitations should be noted. Firstly, this study relied on a voluntary self-reported survey design, which may have affected the response rate and invited bias. Additionally, using a convenience sample means that participants are not representative of undergraduate nursing students and graduates in the country which limits generalization of the findings. Another notable limitation lies in recall bias, particularly for the new graduates who have since transitioned from the university to professional settings. Their retrospective reflections on their learning experiences during their studies could potentially be influenced by their ability to remember their educational experience.

## 5 Conclusion

Despite experiencing emotional and educational challenges during the COVID-19 pandemic, participants in this study felt that their education prepared them for clinical practice. Innovative strategies and unique educational experiences used by educators enhanced participants clinical skills and readiness for practice. It is important, however, that while these strategies assisted, they are no replacement for real world clinical placements which comes with the advantage of human interaction. Successful transition from students to new graduate nurses during the pandemic ensures an adequate and confident nursing workforce that are well-prepared for clinical practice.

## Data availability

Data will be made available on request.

## Additional information

No additional information is available for this paper.

## CRediT authorship contribution statement

**Yasin M. Yasin:** Writing – review & editing, Writing – original draft, Software, Project administration, Methodology, Investigation, Formal analysis, Data curation. **Albara Alomari:** Writing – review & editing, Writing – original draft, Project administration, Methodology, Investigation, Formal analysis, Data curation, Conceptualization. **Wilma ten Ham-Baloyi:** Writing – original draft, Validation, Conceptualization. **Samaneh Alinejad Mofrad:** Writing – original draft, Validation. **Lorna J. Moxham:** Writing – original draft, Formal analysis, Conceptualization. **Elizabeth J. Halcomb:** Writing – original draft, Validation, Supervision, Conceptualization. **Ritin Fernandez:** Writing – review & editing, Writing – original draft, Validation, Supervision, Software, Project administration, Methodology, Formal analysis, Data curation, Conceptualization.

## Declaration of competing interest

The authors declare that they have no known competing financial interests or personal relationships that could have appeared to influence the work reported in this paper.
