# Supplementary material for: Unveiling final-year students and new graduates’ concerns, preparedness, and learning experiences during the pandemic in Qatar: A cross-sectional survey
Source: Heliyon. 2023 Nov 19;9(12):e22337. doi: 10.1016/j.heliyon.2023.e22337 (PMC10724530; doi:10.1016/j.heliyon.2023.e22337)

**DEMOGRAPHICS**

1. What is your age in years?

2. What is your nationality?

3. What is your gender?

Female

Male

I don’t want to answer this question

**MODE OF DELIVERY**

5. Prior to the COVID-19 pandemic, how was the theoretical component of your

nursing program delivered?

Fully face to face (In-person) / On campus

Fully Online / Distance

Combination of Online / face to face (hybrid)

6. Which approach to theoretical learning do you prefer?

Fully face to face (In-person) / On campus

Fully Online

Combination of Online / face to face / hybrid

7. How well do you think your nursing education is preparing you to provide

patient care?

Not at all prepared

Somewhat unprepared

Somewhat prepared

Well prepared

Extremely well prepared

To what degree has the COVID-19 pandemic have an impact on your overall

nursing education?

No impact

Minor impact

Moderate impact

High impact

Very high impact

9. What approach to clinical learning do you prefer?

Placement in health care setting

Face-Face Simulation (Nursing lab session)

Case Scenario

Combination of online / Face to face (hybrid)

Screen-Based (Computerized) Simulation

Other (please specify)

10. Do you feel you have sufficient **skills** to be a competent Registered Nurse?

Yes

No

Unsure

11. Do you feel you have sufficient **knowledge** to be competent Registered

Nurse?

Yes

No

Unsure


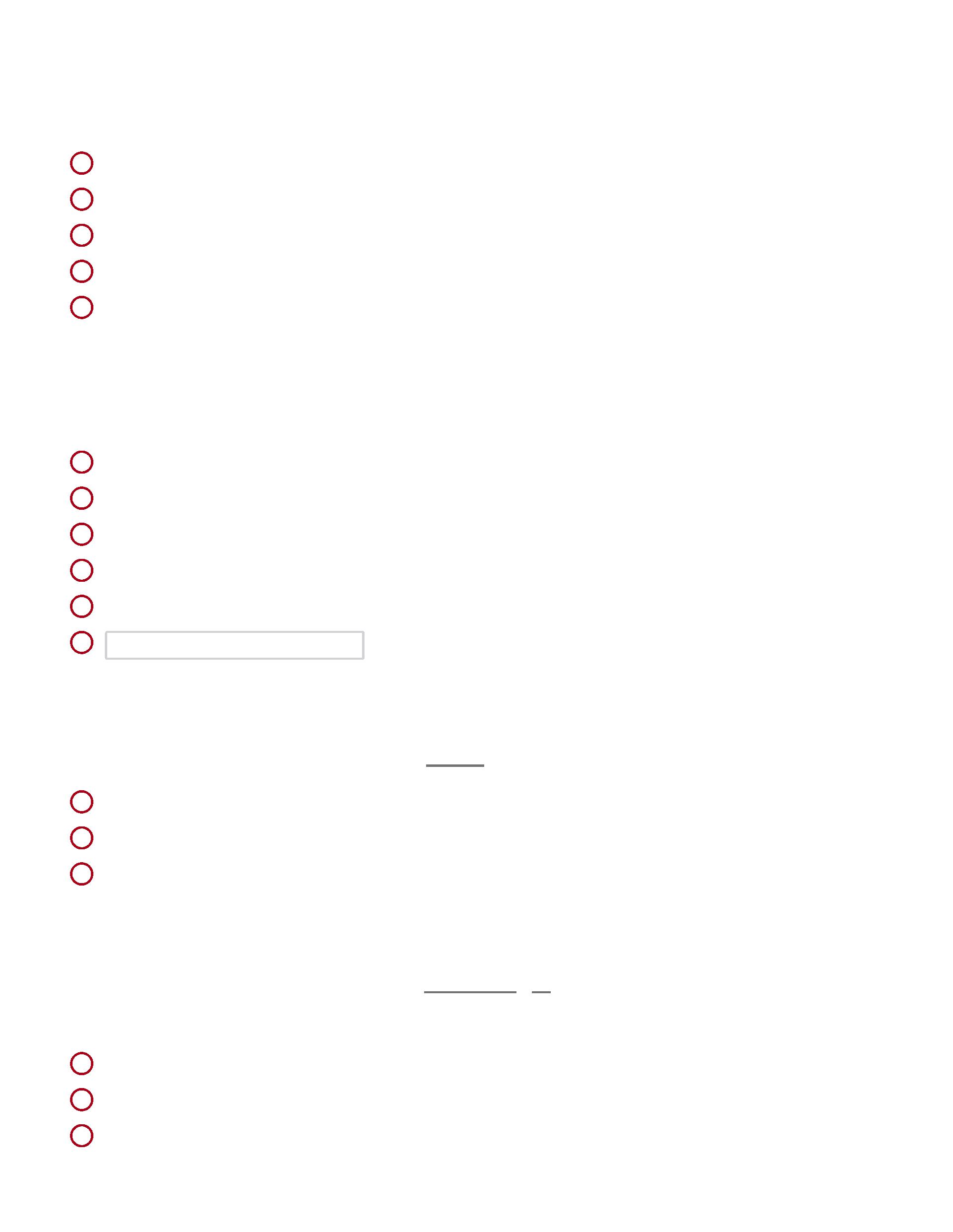


12. Do you feel your undergraduate studies is preparing you to work as a

Registered Nurse?

Yes

No

Unsure

13. Do you feel you are developing sufficient professional values to be a

competent Registered Nurse?

Yes

No

Unsure

14. Has your university provided you with any of the following strategies to

assist with clinical learning during the COVID-19 pandemic (tick all that apply)?

Providing practice kits (e.g. wound care / suturing)

Simulation experiences

Online live chats

Online demonstration of skills

Virtual reality

Increased access to staff

Others

15. Of the strategies you chose in the previous question, Rearrange the top

three that were most helpful to your preparation to enter practice as a

Registered Nurse?


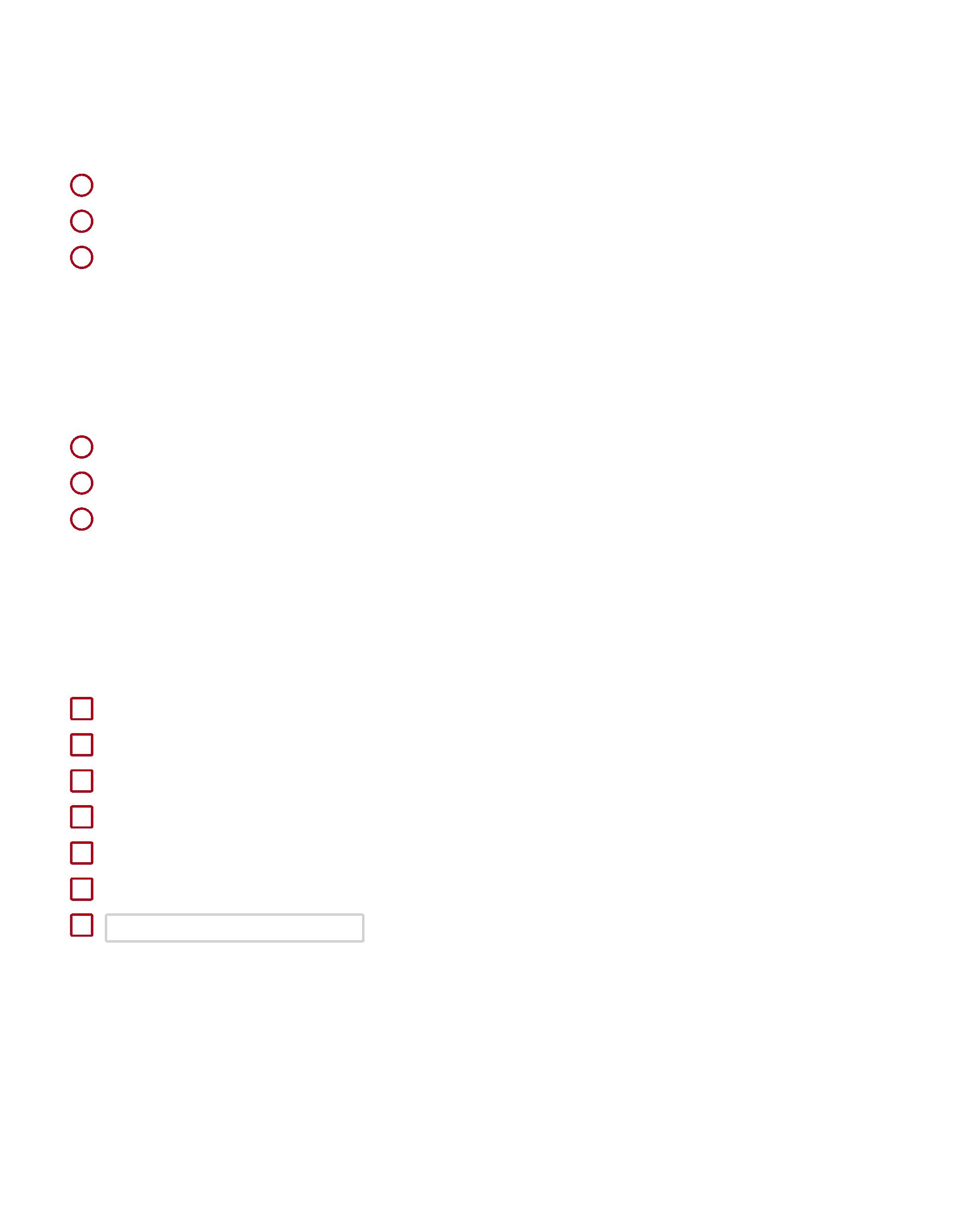


Providing practice kits (e.g. wound care / suturing)

Simulation experiences

Online live chats

Online demonstration of skills

Virtual reality

Increased access to staff

Other

**Preparedness for Clinical Practice**

16. The following statements address specific aspects of your preparedness to

enter clinical practice as a Registered Nurse. Based on your experience during

clinical placements, please indicate by selecting one of the four choices for each

question

Strongly

disagree

Strongly

agree

Disagree

Agree

1. I feel confident communicating with

physicians/doctors

2. I am comfortable communicating

with patients from diverse populations

3. I feel I would be comfortable

delegating tasks to the nursing

Assistant/Assistant in nursing/Nursing

Aides

4. I am having difficulty documenting

care in electronic medical records

5. I have difficulty prioritizing patient

care needs


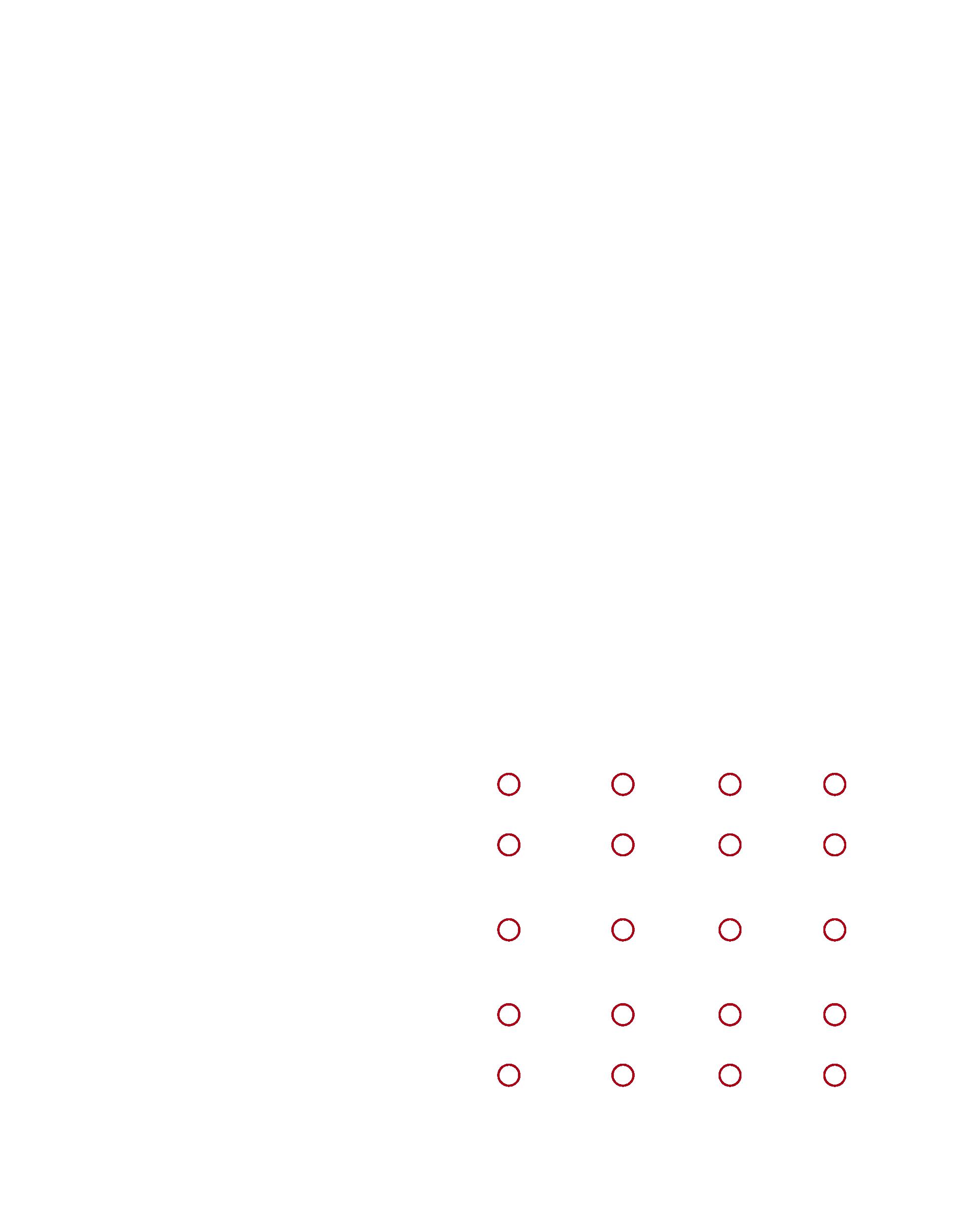


Strongly

disagree

Strongly

agree

Disagree

Agree

6. I feel my preceptor/clinical facilitator

provided feedback about my readiness

to assume an RN role.

7. I am confident in my ability to

problem solve

8. I feel overwhelmed by ethical issues

in my patient care responsibilities

9. I have difficulty recognizing a

significant change in my patient’s

condition

10. I have had opportunities to practice

skills and procedures more than once.

11. I have had opportunities to practice

skills and procedures more than once.

12. I use current evidence to make

clinical decisions

13. I am comfortable communicating

and coordinating care with

interdisciplinary team members

14. Simulations have helped me feel

prepared for clinical practice

15. Writing reflective journals/logs

provided insights into my own clinical

decision-making skills

16. I am comfortable knowing what to

do for a dying patient

17. I am comfortable taking action to

solve problems

18. I feel confident identifying actual or

potential safety risks to my patients

19. I am satisfied with choosing

nursing as a career

20. I feel ready for the professional

nursing role


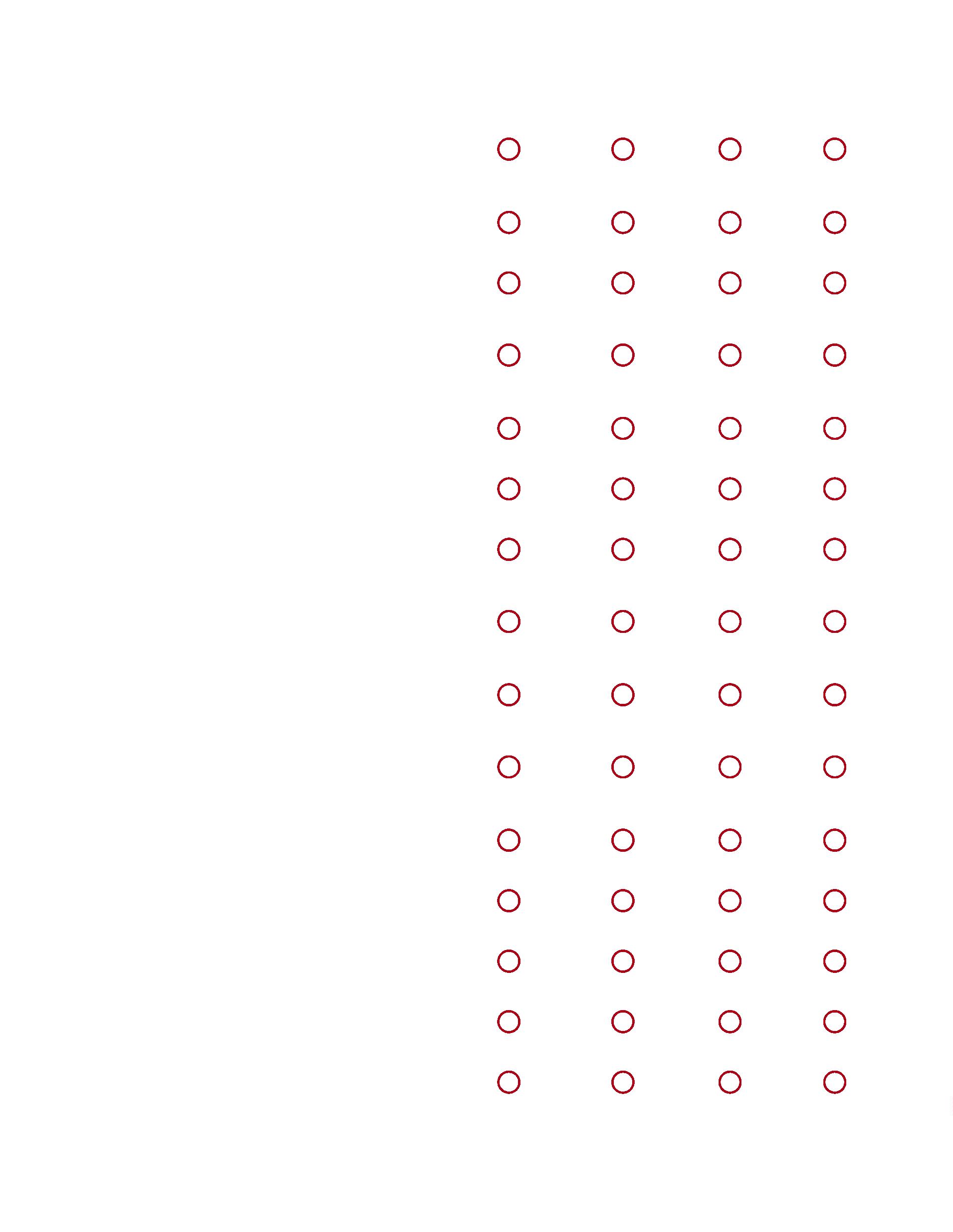


**Concerns**

The following questions ask about your concerns as a student during the

COVID-19 pandemic. Please indicate by selecting one of the five choices for

each question

Neither

agree

Strongly

nor

Strongly

disagree Disagree disagree Agree Agree

1.I was worried about going into the clinical

environment during the pandemic

2.I was worried being in the clinical practice

environment during the pandemic

3.I was worried that I may get COVID-19 if I

attend clinical placement

4. I was worried that my clinical placement will

be cancelled or postponed

5.I was worried that not being able to attend

clinical placement was going to prevent me

from progressing in my degree

6.I was worried that I may not be able to get to

my clinical placement

7.I was worried that not being able to attend

clinical placement was going to result in me

failing my studies

8.I was worried that COVID-19 was going to

have a negative impact on my grades for the

semester

9.I was worried that my ability to submit an

assignment on time was going to be affected

due to consequences of COVID-19 such as

caring for a family member

10.I was worried that the COVID-19 pandemic


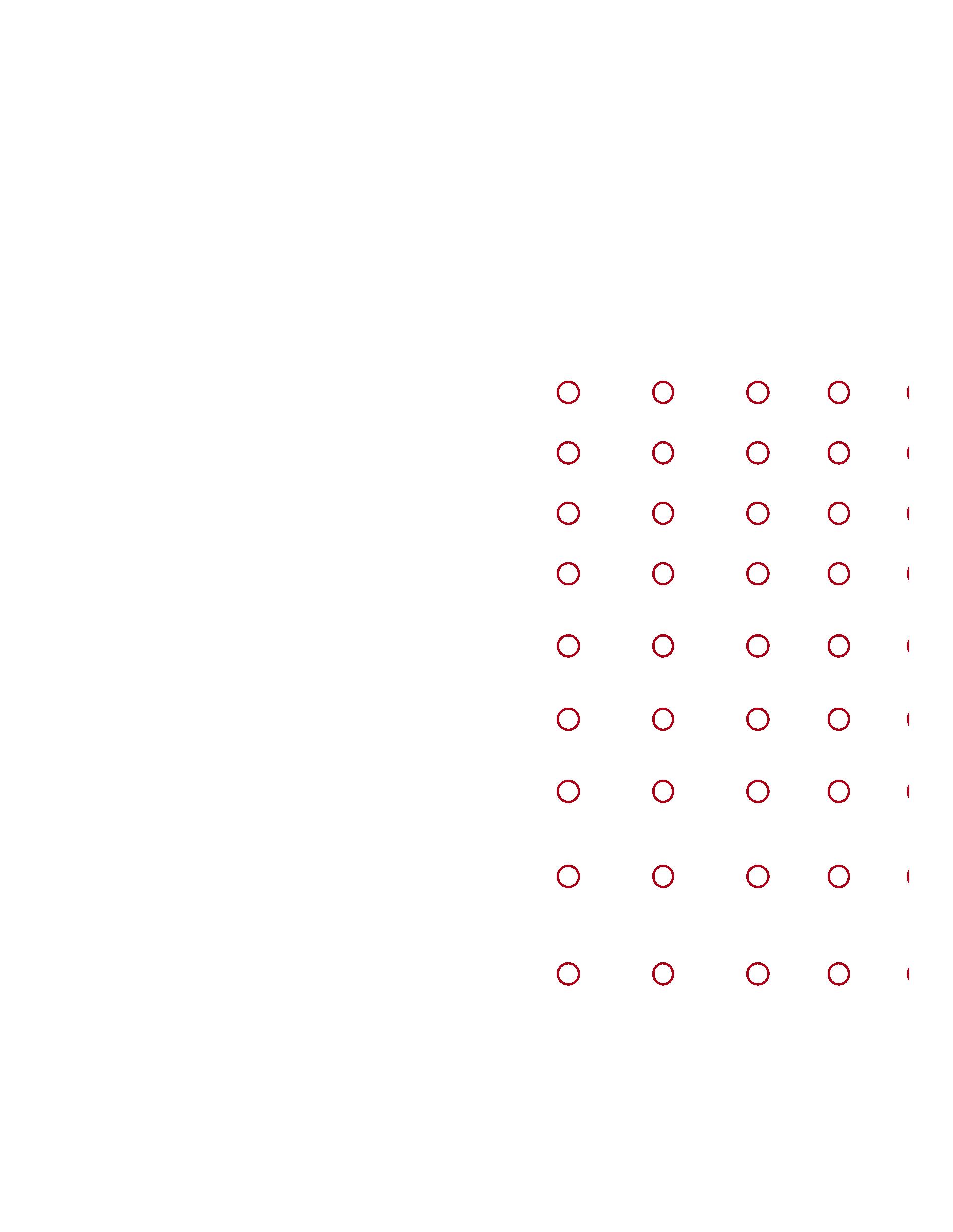


10. Was going to have an impact on my ability to complete my degree

Neither

agree

nor

Strongly

Stro

11.I was worried because I didn't have access

to the Internet to study online

disagree Disagree disagree Agree ag

**General and open ended questions**

18. Please rate the level of stress while studying during the COVID-19

pandemic

0

10 20 30 40 50 60 70 80 90 100

20. Please rate

the level of stress

while studying

during the COVID-

19 pandemic

19. Please indicate the cause of your stress.

No

Yes

Finances

Childcare

Student loads

Living situation

Personal relationships

Job performance

Other (please specify)

20. Please describe the impact that the COVID-19 pandemic has had on your

nursing education

Qualtrics Survey Software

21. List 3 strategies that you are using to manage the impact of the COVID-19

pandemic on your nursing education

22. Please provide us with any additional impacts on your learning as a student

22. What do you think would be most challenging in terms of starting your

career as a Registered Nurse?

24. What is your greatest source of support while studying during the COVID-19

pandemic?

25. What is your opinion about working as a Registered Nurse with

consideration to the COVID-19 pandemic?

I am looking forward to working as a Registered Nurse

I no longer want to work as a Registered Nurse

I am skeptical about working as a Registered Nurse

Other (please specify)

26. Any comments?


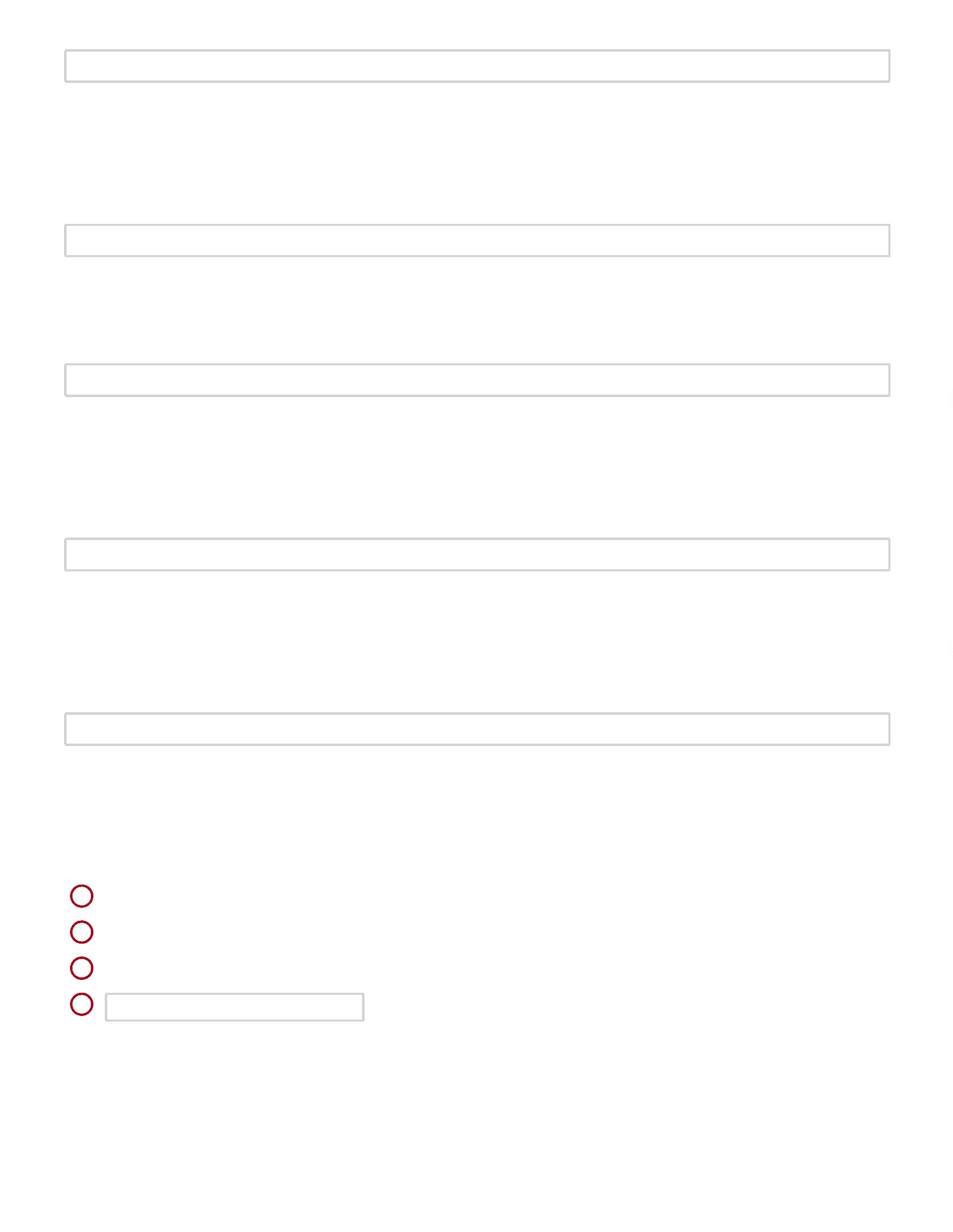

Supplement: Multimedia component 1 [file mmc1.docx]
